# Yoga versus massage in the treatment of aromatase inhibitor-associated knee joint pain in breast cancer survivors: a randomized controlled trial

**DOI:** 10.1038/s41598-021-94466-0

**Published:** 2021-07-21

**Authors:** Chia-Lin Tsai, Liang-Chih Liu, Chih-Ying Liao, Wen-Ling Liao, Yu-Huei Liu, Ching-Liang Hsieh

**Affiliations:** 1 Graduate Institute of Integrated Medicine, China Medical University, No.91, Hsueh-Shih Road, Taichung, 40402 Taiwan; 2 Graduate Institute of Biomedical Sciences, China Medical University, Taichung, Taiwan; 3 Terry Fox Cancer Research Laboratory, Department of Medical Research, China Medical University Hospital, Taichung, Taiwan; 4 Department of Radiation Therapy and Oncology, Department of Health, Taichung Hospital, Executive Yuan, Taichung, Taiwan; 5 Center for Personalized Medicine, China Medical University Hospital, Taichung, Taiwan; 6 Drug Development Centre, China Medical University, Taichung, Taiwan; 7 Department of Medical Genetics and Medical Research, China Medical University Hospital, Taichung, Taiwan; 8 Graduate Institute of Acupuncture Science, China Medical University, No.91, Hsueh-Shih Road, Taichung, 40402 Taiwan; 9 Chinese Medicine Research Centre, China Medical University, Taichung, Taiwan; 10 Department of Chinese Medicine, China Medical University Hospital, Taichung, Taiwan

**Keywords:** Health care, Oncology

## Abstract

Aromatase inhibitors (AIs) are standard adjuvant therapy for postmenopausal women with oestrogen receptor-positive, early-stage, and metastatic breast cancer. Although effective, the risk of falls due to AI-associated knee joint pain significantly increased. The aim of this study was to evaluate the therapeutic effects of yoga and massage on AI-associated knee joint pain. Breast cancer survivors were randomly assigned to a 6-week yoga intervention-2-week rest-6-week massage exposure (Yoga first, n = 30) or a 6-week massage intervention-2-week rest-6-week yoga exposure (Massage first, n = 30). Evaluations of the treatment efficacy were made at baseline, post-intervention, and post-exposure using the Western Ontario and McMaster Universities Osteoarthritis Index (WOMAC) scale, plasma cytokine levels, and changes in meridian energy. The results showed that yoga, superior to massage intervention, significantly reduced AI-associated knee joint pain, as demonstrated by the WOMAC pain score. The yoga intervention improvements were also associated with changes in plasma cytokine levels and meridian energy changes. In conclusion, this study provides scientific evidence that yoga was more effective than massage for reducing AI-associated knee joint pain. Meridian energy changes may provide another scientific, objective, non-invasive way to monitor the therapeutic effects of yoga and investigate another alternative, complementary medicine.

## Introduction

Aromatase inhibitors (AIs) are typical standard adjuvant therapy for postmenopausal women with oestrogen receptor-positive, early-stage, and metastatic breast cancer^1–3^. AIs significantly improve disease-free survival and recurrence of the malignancy^4–6^. Tamoxifen has been used as the primary endocrine therapy for over three decades. Third-generation AIs (anastrozole, letrozole, and exemestane) have a higher potency^7^. Although effective, AIs often increase the risk of various side effects such as mental or memory problems, osteoporosis or bone fractures, AI-associated arthralgia (AIA), and other unclear effects on heart health. AIA is the most common side effect, and it affects 33–74% of patients treated with AIs^8–12^. Although the location is generalized in most cases^13^, a recent study has demonstrated the relationship between arthralgia and falls among AI users, which may attribute to the arthralgia of the knee. According to the survey, 35% of breast cancer survivors reported falls 12–24 months after using an AI. Among women who fell, 2–8% reported seeking medical assistance^14^. The majority of the first pain events developed within 24 months of initiation of treatment (anastrozole 68%, tamoxifen 59%) with a peak occurrence at 6 months (anastrozole 29%, tamoxifen 20%)^13^. Although the events were mild-to-moderate in intensity and did not lead to withdrawal of AI treatment^13^, studies suggest that AIs affect the quality of life with 20–32% of women stopping AI treatment primarily due to AIA^15–17^. As compliance is critical for the success of AI therapy, an intervention to alleviate AIA especially AI-associated knee joint pain, is needed^18^. Pharmacological remedies provide little or no joint symptom relief^19^. Therefore, an evidence-based clinical practice guideline for AIA management is urgently needed.

Potential alternative and complementary interventions such as acupuncture, herbs, vitamins, and physical therapies, such as aerobic/resistance exercise, walking, and yoga show promise for relieving AIA symptoms^19^. A previous meta-analysis revealed positive results with the omega-3 fatty acid^20^. The heterogeneity of physical therapies and the required cohort sizes make it difficult to identify significant benefits^20^. A randomized controlled trial has found that yoga significantly reduced joint pain post-intervention and 90 days later compared to the pre-intervention condition. However, the scale used for joint pain evaluation might not have reflected the joint function^21^. Another single-arm study revealed that yoga significantly improved the worst AI-associated joint pain. However, the sample size was small and again the scale used for pain evaluation might not have reflected the joint function^22^. More scientific evidence for evaluating the effects of yoga intervention is required. Therefore, this randomized, controlled study aims to evaluate the efficacy of yoga compared to massage for relief of AI-associated knee joint pain. The feasibility of the interventions was assessed, and meridian energy was measured as a non-invasive method to monitor the therapeutic effects of yoga. To our knowledge, this is the first assessment of meridian energy to measure the efficacy of yoga.

## Results

### Baseline characteristics of participants

The baseline characteristics of the participants, including age, height, body weight, breast cancer stage, pain time, and exercise habits, are presented in Table 1. Participants' age ranged from 41–70 years, with an average of 53.9 ± 6.4 years. Although a significant difference was found in body weight (*p* = 0.026), body mass index was not affected. There were no other significant differences in the baseline characteristics of the two groups.

**Table 1:** Characteristics of Participants.

	Overall	Yoga first (n = 30)	Massage first (n = 30)	*p* value
**Age (years)**	53.9 ± 6.4 (41‒70)	52.8 ± 6.3 (41‒67)	54.9 ± 6.4 (43‒70)	0.190^a^
**BMI**	23.8 ± 3.7 (17.9‒34.4)	23.0 ± 3.5 (17.9‒32.9)	24.6 ± 3.7 (18.4‒34.4)	0.098^a^
**Height (cm)**	158.8 ± 5.4 (149.0‒169.0)	157.7 ± 5.6 (150.0‒169.0)	159.9 ± 5.2 (149.0‒169.0)	0.111^a^
**Body weight (Kg)**	60.1 ± 9.8 (45.0‒88.0)	57.3 ± 9.1 (45.0‒80.0)	62.9 ± 9.8 (48.0‒88.0)	0.026^a^
**Education**				1.000^b^
High school or less	44 (73.3)	22 (73.3)	22 (73.3)	
College/graduate	16 (26.7)	8 (26.7)	8 (26.7)	
**Marital status**				0.640^b^
Married	55 (91.7)	27 (90.0)	28 (93.3)	
Other	5 (8.3)	3 (10.0)	2 (6.7)	
**Employment status**				0.826^b^
Employed full or part time	22 (36.7)	10 (33.3)	12 (40.0)	
Unemployed	20 (33.3)	11 (36.7)	9 (30.0)	
Retired	18 (30.0)	9 (30.0)	9 (30.0)	
**Stage of breast cancer**				0.841^b^
I	18 (30.0)	8 (26.7)	10 (33.3)	
II	31 (51.7)	16 (53.3)	15 (50.0)	
III	11 (18.3)	6 (20.0)	5 (16.7)	
**Type of treatment**				0.280^b^
Surgery only	1 (1.7)	1 (3.3)	0 (0.0)	
Surgery plus radiation	9 (15.0)	5 (16.7)	4 (13.3)	
Surgery plus chemotherapy	16 (26.7)	5 (16.7)	11 (36.7)	
Surgery plus radiation plus chemotherapy	34 (56.7)	19 (63.3)	15 (50.0)	
**Duration of AI-associated knee joint pain**				0.634^b^
3 ~ 6 months	10 (16.7)	4 (13.3)	6 (20.0)	
6 ~ 12 months	21 (35.0)	9 (30.0)	12 (40.0)	
1 ~ 2 years	14 (23.3)	8 (26.7)	6 (20.0)	
> 2 years	15 (25.0)	9 (30.0)	6 (20.0)	

Data were presented as mean ± SD (range) or N (%).

SD, standard deviation; N, number; AI, aromatase inhibitor.

^a^Independent *t* test.

^b^Chi-square *t *test.

### Attendance and adherence

**Figure 1:**
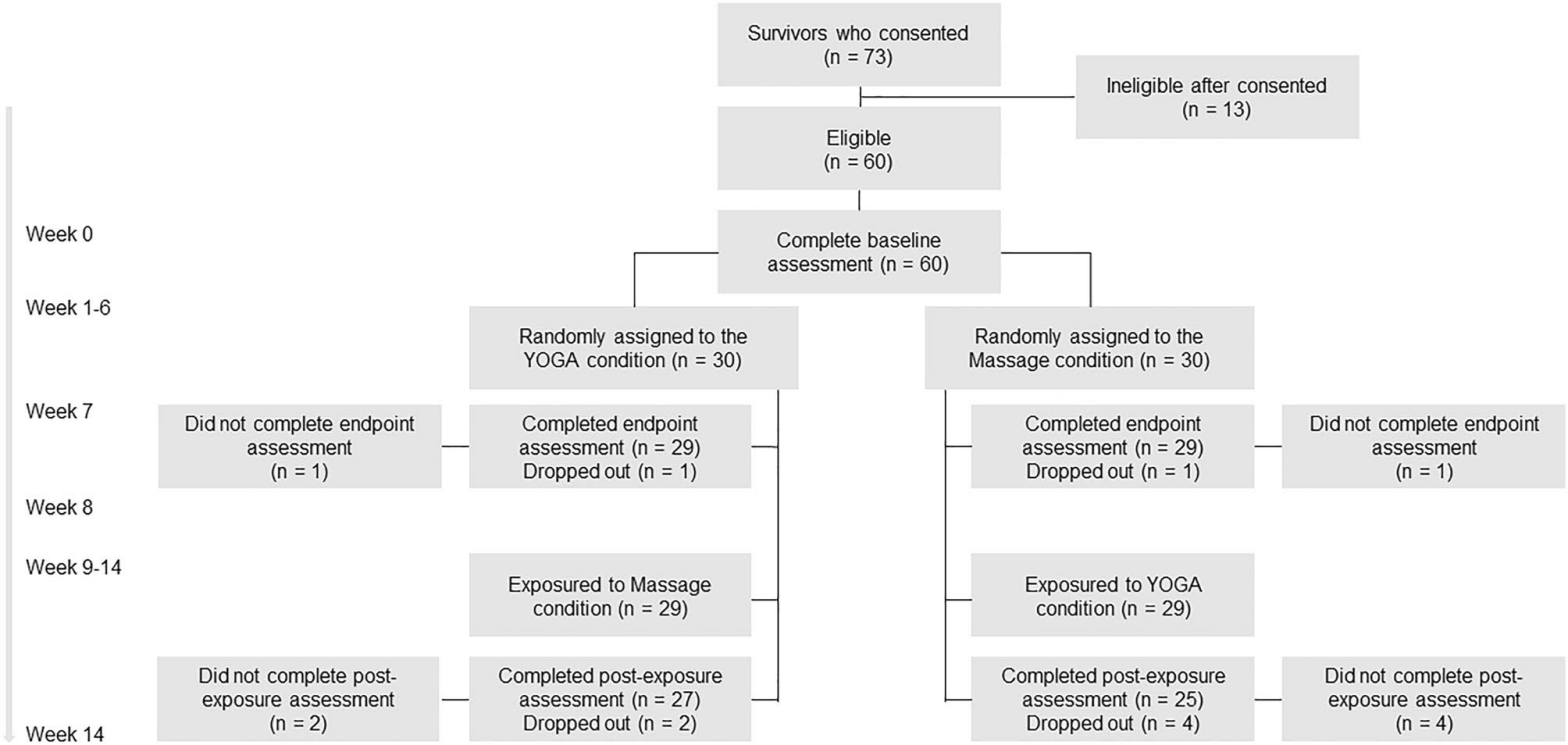
CONSORT diagram showing randomization assignment and participant flow by the group.

Randomization assignment and participant flow by the group were shown in Fig. 1. Written informed consent was obtained from 73 participants, and 60 participants were eligible. Of those who enrolled, two who did not complete baseline assessments and six who did not complete the post-exposure evaluation (13.3%) were excluded from the analysis (Fig. 1). The main reasons for discontinuation were inconvenience (one participant stated family care requirements, two said work requirements, one indicated traffic inconvenience, two had travel scheduled), and personal reasons (one participant had a common cold, one gave no reason). The remaining 52 participants completed all interventions.

### Intervention effects on WOMAC pain scores

Results for the mean WOMAC pain score for the AI-associated knee joint pain are summarized in Table 2. Analysis of covariance analyses (ANCOVA) were not needed to adjusted for baselines due to the homogeneity of regression slopes assumption does not hold (there were covariate by treatment interaction-effect: F(1, 54) = 12.889, *p* = 0.001 for week 7, and F(1, 48) = 9.477, *p* = 0.003 for week 14). The average WOMAC pain score for the AI-associated knee joint pain decreased from 9.3 ± 2.8 at baseline to 4.2 ± 2.2 after yoga intervention in the Yoga first group (*p* = 1.211 × 10^−10^) during the intervention period. The improvements of yoga intervention were sustained for at least one week. However, there was no significant change in the pain scores after massage intervention in the Massage first group (9.7 ± 3.2 to 8.7 ± 4.2, *p* = 0.320). The WOMAC pain scores also decreased to 3.6 ± 2.1 after the 1-week rest and 6-week yoga exposure in the Massage first group, proving no group arrangement bias. However, the WOMAC scores reverted to 8.8 ± 3.1 after the 1-week rest and 6-week massage exposure in the Yoga first group suggested the benefits from yoga intervention could not sustain eight weeks. The results showed that yoga intervention is significantly superior to massage intervention to improve AI-associated knee joint pain. Meanwhile, the benefits of yoga intervention for the AI-associated knee joint pain were sustained for at least one week, but not eight weeks, implying that continual yoga intervention would be critical for the pain management.

**Table 2:** Pain scores of the Western Ontario and McMaster Universities Osteoarthritis Index (WOMAC) outcome comparisons.

Outcome	Yoga first (Mean ± SD)	Massage first (Mean ± SD)	*p* value^a^		*p* value^b^		*p* value^c^
			Yoga First	Massage First	Yoga First	Massage First	
**WOMAC**							
Baseline	9.3 ± 2.8	9.7 ± 3.2					0.606
Week 7	4.2 ± 2.2	8.7 ± 4.2	1.211 × 10^−10^	0.320			3.681 × 10^−6^
Week 14	8.8 ± 3.1	3.6 ± 2.1	0.531	5.863 × 10^−11^	2.280 × 10^−8^	1.168 × 10^−6^	3.881 × 10^−9^

WOMAC, Western Ontario and McMaster Universities Osteoarthritis Index; SD, standard deviation; SE, standard error.

^a^Week 7 vs. baseline and week 14 vs. baseline in groups, paired *t* test.

^b^Week 14 vs. week 7 in groups, paired *t* test.

^c^Baseline, week 7 and week 14 between groups, independent sample *t* test.

### Intervention effects on inflammation

**Figure 2:**
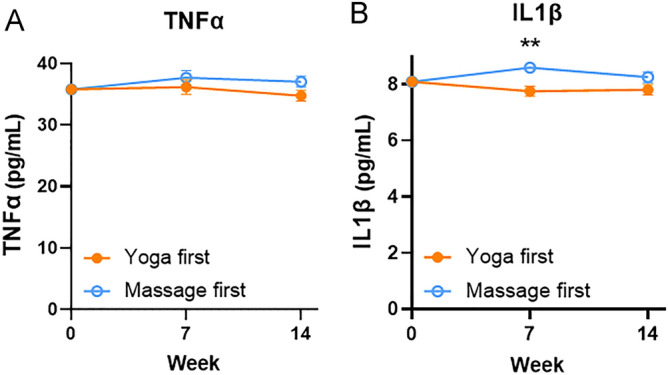
Changes in plasma cytokine levels including tumor necrosis factor alpha (TNF-α) (**A**) and interleukin-1β (IL-1β) (**B**) at baseline, one-week post-intervention (week 7), and post-exposure (week 14) in the Yoga and Massage groups. Results shown are mean ± SE from analysis of covariance tests adjusting for baseline levels. **p < 0.01; ***p < 0.001.

Results for estimated mean plasma cytokines after adjusting for baseline levels are summarized in Fig. 2 and Table 3; unadjusted group means for all outcome variables at each of the three measurement times are available in the supplementary Table 1. The level of IFNγ of all samples was under the detection limit was excluded from statistical analyses. There were no significant differences upon the interventions for TNF-α and IL1-β at baseline, one-week post-treatment, or post-exposure. After adjusting for baseline levels, mean TNF-α levels were not significantly different between groups (week 7 visit: 36.1 ± 1.2 vs. 37.7 ± 1.2, p = 0.360; week 14 visit: 34.7 ± 0.9 vs 37.0 ± 0.9, *p* = 0.082). Although IL1-β levels showed a substantial difference over time between the two groups, the difference appeared due to individual variations. The average IL1-β level was significantly lower in yoga first group compared with massage first group at only week 7 visit (7.8 ± 0.2 vs. 8.6 ± 0.2, *p* = 0.002) but not week 14 visit (7.8 ± 0.2 vs. 8.3 ± 0.2, *p* = 0.113). Those alternations did not mirror the pain score, and could not attribute to a specific intervention. These results indicated that the pain improvement was not significantly associated with levels of inflammatory cytokines such as plasma TNF-α, IFNγ and IL1-β.

**Table 3:** Plasma cytokines and meridian energy outcome comparisons, adjusting for baseline.

Outcome	Estimate	SE	95% CI	*p* value^a^
**TNFα**				
Week 7	− 1.553	1.683	− 4.926 to 1.819	0.360
Week 14	− 2.285	1.289	− 4.875 to 0.305	0.082
**IL1β**				
Week 7	− 0.846	0.260	− 1.367 to − 0.325	0.002
Week 14	− 0.451	0.279	− 1.012 to 0.110	0.113
**Lung meridian (Taiyuan, LU9)**				
Week 7	− 18.864	14.923	− 48.783 to 11.055	0.373
Week 14	5.306	5.941	− 6.633 to 17.244	0.376
**Pericardium meridian (Daling, PC7)**				
Week 7	6.494	5.642	− 4.813 to 17.800	0.255
Week 14	− 6.989	6.148	− 19.345 to 5.367	0.261
**Heart meridian (Shenmen, HT7)**				
Week 7	7.653	5.653	− 3.675 to 18.982	0.181
Week 14	− 4.119	6.877	− 17.940 to 9.701	0.552
**Large intestine meridian (Hegu, LI5)**				
Week 7	8.258	6.891	− 5.552 to 22.068	0.236
Week 14	− 21.746	7.210	− 36.236 to − 7.257	0.004
**Triple energizer meridian (Yangchi, TE4)**				
Week 7	7.797	6.527	− 5.283 to 20.876	0.237
Week 14	− 24.357	7.289	− 39.005 to − 9.701	0.002
**Small intestine meridian (Wangu, SI4)**				
Week 7	3.965	5.685	− 7.428 to 15.359	0.488
Week 14	− 18.843	5.588	− 30.072 to − 7.613	0.001
**Spleen meridian (Taibai, SP3)**				
Week 7	11.919	4.739	2.422 to 21.416	0.015
Week 14	− 18.490	5.536	− 29.615 to − 7.365	0.002
**Liver meridian (Taichong, LR3)**				
Week 7	1.642	5.629	− 9.640 to 12.923	0.772
Week 14	− 25.408	5.183	− 35.823 to − 14.993	1.1 × 10^−5^
**Kidney meridian (Taixi, KI3)**				
Week 7	14.526	4.335	5.838 to 23.213	0.001
Week 14	− 19.993	4.565	− 29.166 to − 10.819	6.244 × 10^−5^
**Stomach meridian (Chongyang, ST42)**				
Week 7	5.489	6.564	− 7.666 to 18.644	0.407
Week 14	− 28.414	6.818	− 42.115 to − 14.713	1.247 × 10^-4^
**Gallbladder meridian (Qiuxu, GB40)**				
Week 7	9.551	4.029	1.475 to 17.626	0.021
Week 14	− 18.331	3.760	− 25.888 to − 10.775	1.184 × 10^−5^
**Bladder meridian (Jinggu, BL65)**				
Week 7	13.462	3.878	5.690 to 21.235	0.001
Week 14	− 23.962	4.238	− 32.478 to − 15.446	7.936 × 10^−7^

TNFα, tumor necrosis factor alpha; IL1β, Interleukin 1 beta; SD, standard deviation; SE, standard error.

^a^*P* value of group effect from analysis of covariance test.

### Intervention effects on meridian energy (ME)

**Figure 3:**
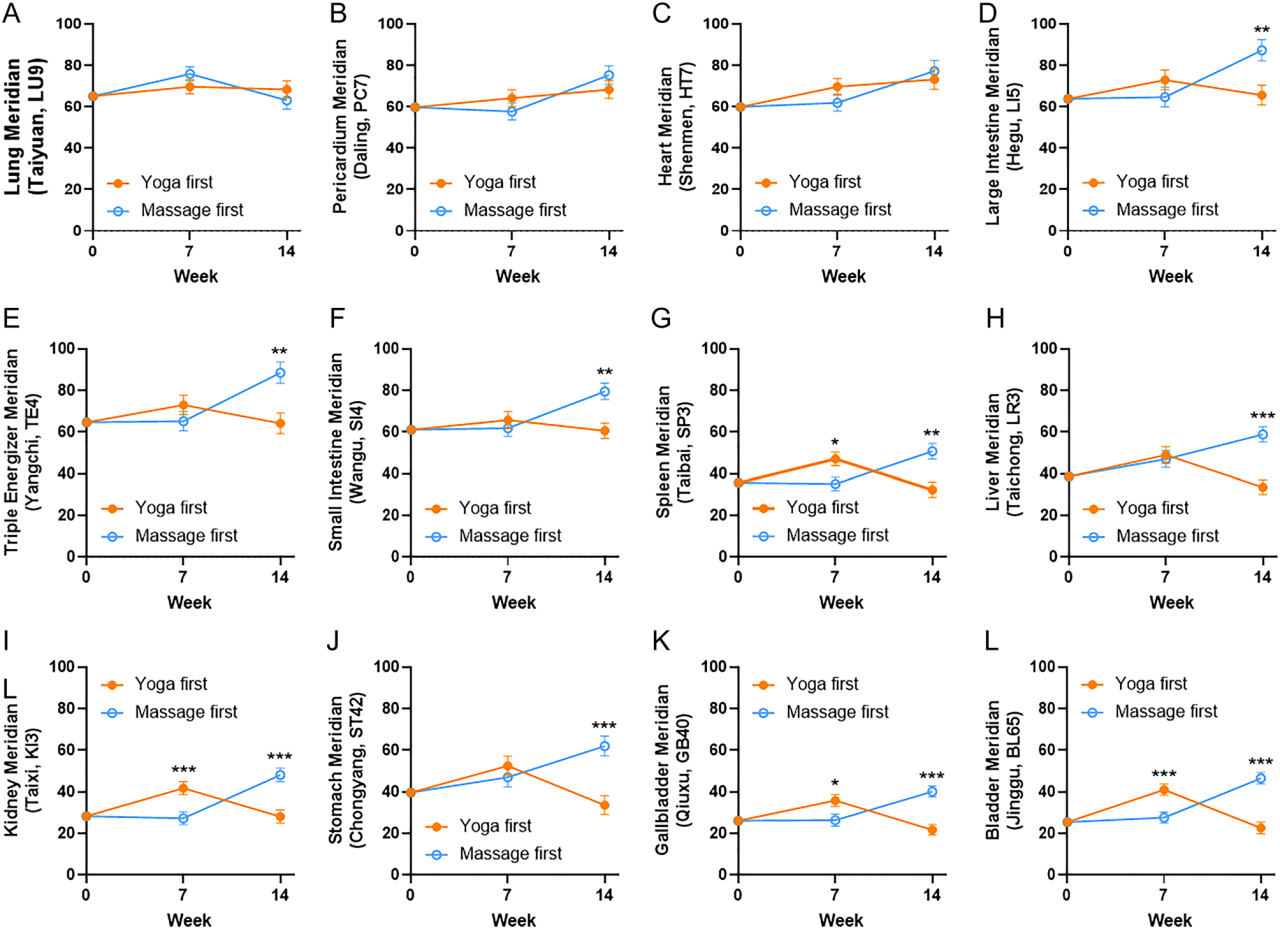
Changes in meridian energy of (**A**) lung meridian (Taiyuan, LU9), (**B**) pericardium meridian (Daling, PC7), (**C**) heart meridian (Shenmen, HT7), (**D**) large intestine meridian (Hegu, LI5), (**E**) triple energizer meridian (Yangchi, TE4), (**F**) small intestine meridian (Wangu, SI4), (**G**) spleen meridian (Taibai, SP3), (**H**) liver meridian (Taichong, LR3), (**I**) kidney meridian (Taixi, KI3), (**J**) stomach meridian (Chongyang, ST42), (**K**) gallbladder meridian (Qiuxu, GB40), (**L**) bladder meridian (Jinggu, BL65) at baseline, one-week post-intervention (week 7), and post-exposure (week 14) in the Yoga and Massage groups. Results shown are mean ± SE from analysis of covariance tests adjusting for baseline levels. *p < 0.05; **, *p* < 0.01; ***, *p* < 0.001.

Results for estimated mean meridian energy after adjusting for baseline levels are summarized in Fig. 3 and Table 3; unadjusted group means for all outcome variables at each of the three measurement times are available in the supplementary Table 2. ANCOVA supported a significant difference favouring yoga over massage for the Week 7 ME enhancement via modulating spleen (SP3, adjusted mean ± SE 47.1 ± 3.3 vs. 35.1 ± 3.3, Yoga first group vs. Massage first group, *p* = 0.015), kidney (KI3, adjusted mean ± SE 41.8 ± 3.1 vs. 27.3 ± 3.1, Yoga first group vs. Massage first group, *p* = 0.001), gallbladder (GB40, adjusted mean ± SE 35.9 ± 2.9 vs. 26.4 ± 2.9, Yoga first group vs. Massage first group, *p* = 0.021) and bladder (BL65, adjusted mean ± SE 41.0 ± 2.7 vs. 27.6 ± 2.7, Yoga first group vs. Massage first group, *p* = 0.001) after adjusted by the baseline. However, the ME of SP3, KI3, GB40, BL65, and additional stomach (ST42) were back after the 1-week rest and 6-week massage exposure in the Yoga first group, mirrored the limited time period of yoga intervention. Not only ME of SP3, KI3, GB40, and BL65, but large intestine (LI5), triple energizer (TE4), Liver (LI5), and ST42 were enhanced. However, the significantly favoring Massage first group over Yoga first group for the Week 14 ME of LI5, TE4, SI4, SP3, LR3, KI3, ST42, GB40, and BL65 after adjusted by the baseline, may also attribute to the carry-out effect (Levene test, *p* < 0.05). No intervention-related safety issues were encountered. These results provide evidence that monitoring meridian energy changes may be a reliable, scientific, and objective non-invasive way to monitor the therapeutic effect of yoga or other alternative and complementary therapies.

### Adverse events

There were no cases of recurrent or metastatic breast cancer detected during the trial. In addition, no intervention-related safety issues were encountered. Events that were potentially attributable to the yoga intervention included five patients with mild dizziness, thirteen with muscle aches, and eight with tiredness. One participant stated all three events.

## Discussion

The current results show that yoga intervention is significantly superior to massage intervention to improve AI-associated knee joint pain, as evaluated by the WOMAC index score. No safety issues were noted with either intervention. While the improvement with yoga intervention can be sustained for at least one week, it cannot be sustained for eight weeks, highlighting the importance of continual yoga intervention. Changes in plasma cytokines and meridian energy provide an additional scientific, objective, non-invasive way to support the therapeutic effects.

In the current study, patient compliance with treatment was good, with 96.7% of participants completing the baseline and post-intervention assessments and 86.7% of participants completing the trial. Although we believe the flexible class arrangements increased the participation rate compared to other randomized controlled trials, a few participants still dropped out due to inconveniences, including class time and location. Decentralized community yoga classes or home-based online yoga classes provided by instructors after an initial in-person class may help improve participant compliance. A greater understanding of the attitudes and barriers to yoga practice will help provide better yoga programs aligned to the population's needs. The social support status, fatigue, and depressive symptoms were not part of the inclusion criteria. However, beneficial observations were seen for fatigue, vitality, and insomnia, all commonly reported by patients taking AIs. These factors may warrant further investigation.

Although a previous study showed that yoga practice substantially reduced inflammatory cytokines^23^, only a similar trend of IL1-β, but not TNF-α was not found in our trial. In the previous study, lipopolysaccharide-stimulated production of interleukin-6 (IL-6), TNF-α, and IL-1β from isolated peripheral blood mononuclear cells was measured by using an electrochemiluminescence method^23^, which may reflect anti-microbial infection abilities. However, in current study, the levels of plasma TNF-α, IFN-γ, and IL1-β were measured by using ELISA, reflecting the physiological conditions. Besides, the detection time point, population differences, and personal differences may account for these results. Exercise training reduces inflammation are sparse and inconsistent^24,25^. Weight loss may be crucial for the change in inflammatory cytokines^24,25^. Further study is warranted to assess the impact of yoga on cytokines.

Measurement of meridian energy changes was used as a novel method to evaluate the efficacy of study interventions. Meridian energy theory is based on traditional Chinese Medicine (TCM) and remains debated in Western medicine^26^. The meridian system is a series of pathways that conduct energy, known as "qi", throughout the body. The meridians are associated with two balanced forces, "yin" and "yang". Disruption to the flow of energy in these pathways is believed to be the cause of illness. The meridian energy analysis device (MEAD), with reproducibility of 93.2%, has been recognized as a reliable complementary device for evaluating the efficacy of traditional Chinese medicine (TCM) therapy, related sympathetic conditions, and tracing the meridian flow^27,28^. A review with recommendations for clinical trials has been suggested^29^. We designed the studies based on this advice and performed measurements that followed the manufacturer's instructions to maximize the reliability.

It was noted that there were participants with a relative meridian energy deficiency (less than 50 μA) at the spleen (SP3), liver (LR3), kidney (KI3), stomach (ST42), gallbladder (GB40), and bladder (BL65), suggesting that AI-associated knee joint pain in breast cancer survivors has a deficiency syndrome through these meridians. According to the current results, the yoga intervention superior over massage intervention via improving spleen (SP3), kidney (KI3), gallbladder (GB40) and bladder (BL65) energy. According to TCM theory, the yang meridians of the stomach (ST42), gallbladder (GB40) and bladder (BL65) distribute energy from the head, across the trunk, along the front, lateral, and back sides of the leg to the foot, repectively. The yin meridians of the spleen (SP3), liver (LR3), and kidney (KI3) distribute energy from the foot, up the inner side of the leg, across the front of the abdomen, and chest to the head. The meridians that demonstrated improvement with yoga intervention contribute mainly to the leg, which coincides with the improved knee pain and WOMAC score results. The bladder meridian (BL65) is the longest meridian and the largest detoxification and dehumidification channel of the human body. Improvement of BL65 energy will help to eliminate the phlegm and waste in the body. When BL65 energy is lower, moisture and toxins could not be excreted from the body, which will easily lead to blood stasis and cause many physical pains. Moreover, the KI3 and the BL65 are mirrored. Improvement of the BL65 is believed to increase the metabolism and excretion of moisture and toxins in the body through urine, by which reduce body pain. To the best of our knowledge, this is the first study to link meridian energy to the evaluation of yoga intervention. The energy increased by yoga intervention is thought to be "qi", which has been implied as mitochondria^30^. However, minimal resources have been invested in understanding mitochondrial biology and genes due to the lack of feasible methods and facilities. A better understanding of mitochondria may bridge the gap between Western medicine and TCM.

The limitation of the current study includes the existence of the carry-out effect. Although each subject has received two different treatments, yoga and massage, after random entry into the trial, with yoga first group receives yoga and then massage treatment, and the massage first group massage first and then yoga treatment. A more appropriate washout period or more frequent measurements may help to identify the real treatment effect.

In conclusion, the current results show that yoga intervention reduces AI-associated knee joint pain in breast cancer survivors, as demonstrated by the reduced WOMAC score. In addition, it supports the feasibility and potential efficacy of yoga intervention and provides scientific evidence that yoga is more effective than massage for reducing AI-associated knee joint pain. Measurement of the meridian energy change using a reliable device might be a potential, scientific, objective, and non-invasive way to monitor the therapeutic effects of yoga as well as any other alternative, complementary medicine. Future studies with larger cohorts are warranted.

## Methods

### Study design

This randomized controlled trial (RCT) compared a yoga intervention with a home-based massage intervention. Breast cancer survivors were randomly assigned to a 6-week yoga intervention-2-week rest-6-week massage exposure (Yoga first, n = 30) or a 6-week massage intervention-2-week rest-6-week yoga exposure (Massage first, n = 30). Questionnaires and blood samples were collected at baseline, one-week post-intervention, and one-week post-exposure. The study followed the Declaration of Helsinki and was approved by the Medical Ethics Committee of China Medical University Hospital (CMUH107-REC3-102, approved on 06/09/2018) and retrospectively registered at ClinicalTrials.gov (NCT03956875, recorded on 21/05/2019). All participants provided written informed consent.

### Participants

Sixty patients with stage I–III breast cancer stage (mean age 53.9 ± 6.4 y; range 41–70 years at enrolment) from the China Medical University Hospital were enrolled from 06/09/2018 to 05/09/2019. Participants were recruited through oncologist referrals, community print advertisements and announcements, and breast cancer groups and events. Patients were required to have diagnosed stage I–III breast cancer at the time of examination, be currently taking AIs, and have a history of AI-associated knee joint pain for at least 3 months. Patients were excluded if suffering from multiple non-breast cancer-related pain sources, such as visceral pain and trauma pain. Other exclusions included: patients with addictive drug abusers; patients who might become pregnant or had delivered a baby within 3 months; patients unable to understand or give informed consent due to severe mental illness, an unstable mental state, severe hearing or visual impairment, or dementia; patients unable to practice yoga due to severe limb oedema, skin damage, and spinal disease; patients who had participated in yoga in the past month, or with routine exercise for more than 90 min per week; and patients with stage IV metastatic breast cancer.

### Sample size estimation

The sample size was estimated based on detecting meaningful differences in primary endpoints with 80% power and a one-sided 5% significance level using G*Power 3.1^31,32^. Effect sizes were based on a previously published study^23^ and data we collected from earlier experience. The estimated requirement was 26 patients per group, with 10% attrition expected, resulting in 30 patients per group. The Consolidated Standards of Reporting Trials (CONSORT) checklist is provided in Fig. 1 (http://www.consort-statement.org/consort-2010)^33^.

### Randomization

Sixty participants who met the inclusion criteria and provided written informed consent were randomly divided into the Yoga first and Massage first groups, with 30 in each group. Random numbers were produced through randomization using Excel version 16.0 (Microsoft Office, Redmond, WA, USA). Dr. Chia-Lin Tsai performed simple randomization. The random numbers were placed into sealed envelopes. A serial number was assigned to each envelope by the yoga instructors following the allocation sequence of the randomized numbers. Each envelope was opened sequentially according to the admission sequence of participants at the research centre. The number inside the envelope determined the group the participant was allocated. The study included an intervention period, a washout, and an exposure period. The Yoga first group performed yoga exercises two times per week for six weeks (intervention), rested for two weeks, and then completed six weeks of home-based massage (exposure). The Massage first group performed six weeks of home-based massage (intervention), rested for two weeks, then performed yoga twice a week for six weeks (exposure).

### Interventions

Yoga classes were led by two certified yoga instructors and were held every morning in a private classroom specifically for study participants, providing participants flexibility in scheduling the twice weekly, sixty-minutes class time. The class format included Mindfulness yoga, the traditional Hatha yoga modified for different conditions and symptoms^34^. Sequences included standing poses, forward extensions, inversions, backbends, and twists. Participants were assisted in adopting a series of postures and using breathing techniques designed by the instructors. Assistance included ad hoc props such as blocks, pillows, or blankets when required.

### Assessment of pain

The pain section of the Chinese version of the Western Ontario and McMaster Universities Osteoarthritis Index (WOMAC) scale^35,36^ was used to evaluate intervention outcomes. The pain scale contained 5 items that were scored on a scale of 0–4 (0 = none, 1 = slight, 2 = moderate, 3 = very, 4 = extremely). All assessments were evaluated by the same well-trained physician who was blinded to the study randomization to ensure reliability and validity.

### Measurement of meridian energy

The energy of the twelve regular meridians^37^ was measured with a meridian energy analysis device (MEAD ME-20, Medpex. Inc., Taichung, Taiwan) according to the manufacturer's instructions. A relative current less than 50 (A was considered "deficiency" syndrome and greater than 90 (A was considered "excess" syndrome. All measurements were recorded twice on each side by the same well-trained physician who was blinded to study randomization to ensure reliability and validity.

### Plasma cytokine measurement

All separated plasma samples were stored at − 80 °C until analysis. All measurements were performed using the TNF alpha Human Uncoated ELISA Kit (Cat # 88–7346-88), IFN gamma Human Uncoated ELISA Kit (Cat # 88–7316-88), and IL-1 beta Human Uncoated ELISA Kit (Cat # 88–7261-88) obtained from Thermo Fisher Scientific (Waltham, MA, USA) according to the manufacturer's instructions.

### Statistical analyses

Statistical analyses were performed using the PASW Statistics 18.0 software (SPSS Inc., Chicago, IL, USA). Chi-square, paired *t* tests, idependent sample t test, analysis of variance, or ANCOVA were used to evaluate the associations between groups when appropriate.

## Supplementary Information

Supplementary Table 1.

Supplementary Table 2.
