# Supplementary material for: Yoga versus massage in the treatment of aromatase inhibitor-associated knee joint pain in breast cancer survivors: a randomized controlled trial
Source: Sci Rep. 2021 Jul 21;11:14843. doi: 10.1038/s41598-021-94466-0 (PMC8295273; doi:10.1038/s41598-021-94466-0)
Supplement: Supplementary file 1 — Supplementary Table 1. [file 41598_2021_94466_MOESM1_ESM.docx]

**Supplementary Table 1. Comparisons of plasma cytokines**

| Outcome |  | Yoga first  (Mean ± SD) | Massage first  (Mean ± SD) | p value ^a^ |  | p value ^b^ |  | p value ^c^ |
| --- | --- | --- | --- | --- | --- | --- | --- | --- |
|  |  |  |  | Yoga  First | Massage  First | Yoga  First | Massage  First |  |
| TNFα | Baseline | 37.0 ± 5.2 | 35.1 ± 4.1 |  |  |  |  | 0.108 |
|  | Week 7 | 36.4 ± 5.6 | 36.2 ± 9.7 | 0.645 | 0.110 |  |  | 0.939 |
|  | Week 14 | 35.2 ± 4.8 | 36.5 ± 5.6 | 0.185 | 0.275 | 0.417 | 0.582 | 0.387 |
| IFNγ ^d^ | Baseline | 1.9 ± 0.9 | 2.2 ± 1.3 |  |  |  |  |  |
|  | Week 7 | 1.6 ± 0.7 | 2.2 ± 0.9 |  |  |  |  |  |
|  | Week 14 | 1.4 ± 0.6 | 2.3 ± 1.0 |  |  |  |  |  |
| IL1β | Baseline | 7.8 ± 0.6 | 8.4 ± 1.4 |  |  |  |  | 0.030 |
|  | Week 7 | 7.5 ± 1.1 | 8.8 ± 1.4 | 0.258 | 0.278 |  |  | 2.535 × 10^-4^ |
|  | Week 14 | 7.6 ± 1.4 | 8.5 ± 1.2 | 0.520 | 0.931 | 0.787 | 0.317 | 0.025 |

Abbreviation: TNFα, tumor necrosis factor-alpha; IFNγ, Interferon-gamma; IL1β, Interleukin 1-beta; SD, standard deviation; SE, standard error.

a. Week 7 vs. baseline and week 14 vs. baseline in groups, paired t test.

b. Week 14 vs. week 7 in groups, paired t test.

c. Baseline, week 7 and week 14 between groups, independent sample t test.

d. The level of IFNγ of all samples was under the detection limit.
