# Supplementary material for: Yoga versus massage in the treatment of aromatase inhibitor-associated knee joint pain in breast cancer survivors: a randomized controlled trial
Source: Sci Rep. 2021 Jul 21;11:14843. doi: 10.1038/s41598-021-94466-0 (PMC8295273; doi:10.1038/s41598-021-94466-0)
Supplement: Supplementary file 2 — Supplementary Table 2. [file 41598_2021_94466_MOESM2_ESM.docx]

**Supplementary Table 2. Meridian Energy Outcome Comparisons**

| Meridian Energy |  | Yoga first  (Mean ± SD) | Massage first  (Mean ± SD) | p value ^a^ |  | p value ^b^ |  | p value ^c^ |
| --- | --- | --- | --- | --- | --- | --- | --- | --- |
|  |  |  |  | Yoga  First | Massage  First | Yoga  First | Massage  First |  |
| Lung Meridian  (Taiyuan, LU9) | Baseline | 67.1 ± 25.9 | 61.4 ± 22.3 |  |  |  |  | 0.363 |
|  | Week 7 | 70.9 ± 22.7 | 75.0 ± 21.5 | 0.555 | 0.020 |  |  | 0.478 |
|  | Week 14 | 70.6 ± 26.6 | 60.6 ± 24.1 | 0.620 | 0.893 | 0.966 | 0.024 | 0.162 |
| Pericardium Meridian  (Daling, PC7) | Baseline | 59.6 ± 25.4 | 58.1 ± 22.5 |  |  |  |  | 0.816 |
|  | Week 7 | 64.1 ± 21.7 | 57.6 ± 26.7 | 0.460 | 0.935 |  |  | 0.310 |
|  | Week 14 | 69.1 ± 26.6 | 74.2 ± 24.7 | 0.171 | 0.014 | 0.445 | 0.022 | 0.477 |
| Heart Meridian  (Shenmen, HT7) | Baseline | 58.1 ± 25.8 | 60.0 ± 22.1 |  |  |  |  | 0.766 |
|  | Week 7 | 68.7 ± 22.4 | 62.7 ± 27.4 | 0.097 | 0.670 |  |  | 0.364 |
|  | Week 14 | 73.7 ± 29.7 | 76.7 ± 27.4 | 0.038 | 0.016 | 0.482 | 0.068 | 0.711 |
| Large Intestine Meridian  (Hegu, LI5) | Baseline | 68.6 ± 36.5 | 57.0 ± 28.7 |  |  |  |  | 0.178 |
|  | Week 7 | 75.5 ± 28.9 | 62.0 ± 31.0 | 0.424 | 0.521 |  |  | 0.093 |
|  | Week 14 | 69.4 ± 31.5 | 82.9 ± 24.6 | 0.927 | 0.001 | 0.455 | 0.009 | 0.092 |
| Triple Energizer Meridian  (Yangchi, TE4) | Baseline | 65.9 ± 36.1 | 60.7 ± 33.0 |  |  |  |  | 0.563 |
|  | Week 7 | 74.4 ± 29.0 | 63.8 ± 33.8 | 0.320 | 0.720 |  |  | 0.204 |
|  | Week 14 | 67.0 ± 31.9 | 85.5 ± 27.3 | 0.897 | 0.004 | 0.368 | 0.013 | 0.030 |
| Small Intestine Meridian  (Wangu, SI4) | Baseline | 64.1 ± 28.2 | 55.7 ± 26.2 |  |  |  |  | 0.233 |
|  | Week 7 | 67.8 ± 24.0 | 60.0 ± 26.1 | 0.596 | 0.531 |  |  | 0.240 |
|  | Week 14 | 63.6 ± 26.7 | 76.2 ± 19.6 | 0.945 | 0.002 | 0.543 | 0.014 | 0.061 |
| Spleen Meridian  (Taibai, SP3) | Baseline | 41.8 ± 22.7 | 28.8 ± 17.9 |  |  |  |  | 0.017 |
|  | Week 7 | 50.3 ± 21.5 | 31.9 ± 17.6 | 0.144 | 0.507 |  |  | 0.001 |
|  | Week 14 | 38.0 ± 22.1 | 44.6 ± 22.5 | 0.531 | 0.005 | 0.039 | 0.024 | 0.293 |
| Liver Meridian  (Taichong, LR3) | Baseline | 41.3 ± 30.7 | 34.7 ± 24.7 |  |  |  |  | 0.360 |
|  | Week 7 | 50.8 ± 25.0 | 45.6 ± 26.2 | 0.200 | 0.105 |  |  | 0.445 |
|  | Week 14 | 37.3 ± 27.2 | 54.8 ± 19.4 | 0.604 | 0.002 | 0.058 | 0.153 | 0.011 |
| Kidney Meridian  (Taixi, KI3) | Baseline | 29.3 ± 21.2 | 26.0 ± 20.0 |  |  |  |  | 0.538 |
|  | Week 7 | 42.6 ± 19.5 | 26.6 ± 19.0 | 0.016 | 0.915 |  |  | 0.002 |
|  | Week 14 | 30.1 ± 22.0 | 45.8 ± 20.3 | 0.888 | 0.001 | 0.029 | 0.001 | 0.011 |
| Stomach Meridian  (Chongyang, ST42) | Baseline | 44.2 ± 29.3 | 33.2 ± 26.5 |  |  |  |  | 0.133 |
|  | Week 7 | 54.7 ± 23.7 | 44.9 ± 29.3 | 0.138 | 0.115 |  |  | 0.164 |
|  | Week 14 | 38.1 ± 28.9 | 57.1 ± 22.6 | 0.431 | 0.001 | 0.022 | 0.095 | 0.011 |
| Gallbladder Meridian  (Qiuxu, GB40) | Baseline | 26.6 ± 20.4 | 24.6 ± 18.2 |  |  |  |  | 0.700 |
|  | Week 7 | 36.4 ± 18.3 | 25.9 ± 17.7 | 0.057 | 0.780 |  |  | 0.031 |
|  | Week 14 | 23.6 ± 19.4 | 38.1 ± 18.0 | 0.579 | 0.008 | 0.014 | 0.016 | 0.008 |
| Bladder Meridian  (Jinggu, BL65) | Baseline | 32.5 ± 21.6 | 18.7 ± 13.4 |  |  |  |  | 0.004 |
|  | Week 7 | 45.2 ± 18.3 | 23.4 ± 16.8 | 0.019 | 0.235 |  |  | 1.621 × 10 ^-5^ |
|  | Week 14 | 29.7 ± 19.9 | 38.9 ± 18.9 | 0.603 | 2.477 × 10 ^-5^ | 0.004 | 0.002 | 0.094 |

Abbreviation: SD, standard deviation; SE, standard error.

a. Week 7 vs. baseline and week 14 vs. baseline in groups, paired t test.

b. Week 14 vs. week 7 in groups, paired t test.

c. Baseline ,week 7 and week 14 between groups, idependent sample t test.
